# Dendritic Cell Migration to Skin-Draining Lymph Nodes Is Controlled by Dermatan Sulfate and Determines Adaptive Immunity Magnitude

**DOI:** 10.3389/fimmu.2018.00206

**Published:** 2018-02-08

**Authors:** Reza Nadafi, Jasper J. Koning, Henrike Veninga, Xanthi N. Stachtea, Tanja Konijn, Antonie Zwiers, Anders Malmström, Joke M. M. den Haan, Reina E. Mebius, Marco Maccarana, Rogier M. Reijmers

**Affiliations:** ^1^Department of Molecular Cell Biology and Immunology, VU University Medical Center, Cancer Center Amsterdam, Amsterdam, Netherlands; ^2^Department of Experimental Medical Science, Lund University, Lund, Sweden

**Keywords:** skin–draining lymph node, adaptive immunity, Dse, dendritic cells, dermatan sulfate epimerase, collagen, antigen, Ehlers–Danlos syndrome

## Abstract

For full activation of naïve adaptive lymphocytes in skin-draining lymph nodes (LNs), presentation of peptide:MHC complexes by LN-resident and skin-derived dendritic cells (DCs) that encountered antigens (Ags) is an absolute prerequisite. To get to the nearest draining LN upon intradermal immunization, DCs need to migrate from the infection site to the afferent lymphatics, which can only be reached by traversing a collagen-dense network located in the dermis of the skin through the activity of proteolytic enzymes. Here, we show that mice with altered collagen fibrillogenesis resulting in thicker collagen fibers in the skin display a reduced DC migration to the draining LN upon immune challenge. Consequently, the initiation of the cellular and humoral immune response was diminished. Ag-specific CD8+ and CD4+ T cells as well as Ag-specific germinal center B cells and serum immunoglobulin levels were significantly decreased. Hence, we postulate that alterations to the production of extracellular matrix, as seen in various connective tissue disorders, may in the end affect the qualitative outcome of adaptive immunity.

## Introduction

Lymph nodes (LNs) are strategically located secondary lymphoid organs that drain nearly all tissues of the body, including the skin as the largest organ, to collect foreign soluble and particulate antigens (Ags) through a lymphatic vasculature (1, 2). The collection of Ags is essential for a controlled and rapid initiation of a robust and Ag-specific adaptive immune response (2–4). Ag-containing lymph will flow *via* the afferent vessels into the sinuses and a collagen-rich conduit system of the draining LN, produced by lymphoid stromal cells (5–7). LN-resident dendritic cells (DCs) sample the conduits and sinuses for Ags and present these as peptide:MHC complexes for initial activation of naïve T cells (1, 2). Next, newly arriving tissue-resident DCs sequentially present peptide:MHC complexes to Ag-primed naïve T cells, essential for full activation and successive proliferation. Importantly, when migration of distant DCs was prevented upon rapid removal of the injection site, activation of T cells was significantly diminished and the cellular immunity severely hampered (1, 2).

To reach the afferent lymphatics, DCs from the skin need to pass a barrier in the dermis, consisting of a dense but highly organized and balanced network of collagens, which requires the obligatory activity of metalloproteinases (8). Interestingly, when the collagen content in the skin was reduced by 50%, which was found in mice deficient for secreted protein acidic and rich in cysteine (*Sparc*), DCs displayed an accelerated emigration from the skin resulting in an abnormally rapid T cell activation (9). These findings support a model in which the composition and density of the dermal extracellular matrix (ECM) determines the efficiency of DC emigration and subsequent immune activation in skin-draining LNs (sdLNs).

Notably, collagen fibrillogenesis is strictly controlled by many ECM proteins, including small leucine-rich proteoglycans that form essential interfibrillar bridges through chondroitin/dermatan sulfate (CS/DS) glycosaminoglycans (GAGs) attached to a core protein (10, 11). To execute their biological functions, CS/DS GAGs undergo crucial and extensive modification by Golgi apparatus-located biosynthetic enzymes (12). Of these enzymes, DS-epimerase 1 in close conjunction with DS-4-O-sulfotransferase 1 (D4st1 or Chst14) are key in the transformation of CS to hybrid CS/DS chains (13). Downregulation or loss of either enzyme changes the composition of the CS/DS GAGs, affecting biological functions (12). Remarkably, next to DS-epimerase 1, a second epimerase exists, DS-epimerase 2, which has overlapping functions; however, these biosynthetic enzymes have been shown to display striking tissue-specific distribution. Whereas DS-epimerase 1 is the main epimerase in most tissues of the body including the skin (14), DS-epimerase 2 is most prominently present in the brain (15).

Although both epimerases are ubiquitously expressed, each isoform specifically contributes to spatially distinct CS/DS GAGs. As a consequence, mice only lacking DS-epimerase 1 display an altered collagen structure in the skin, which could not be compensated for by DS-epimerase 2 (14). Similarly, this non-redundancy has recently been observed in humans with a homozygous *DSE* missense mutation (16). These patients were classified as a subtype of Ehlers–Danlos syndrome, a connective tissue disorder. Importantly, the Ehlers–Danlos syndromes represent a heterogeneous group of diseases, which are well known for their fragility of the soft connective tissues, including the skin. Therefore, we set out to study the effect of DS epimerase-1 (*Dse*) deficiency, in mice, on the adaptive immune response in sdLNs initiated after intradermal immunization.

## Materials and Methods

### Mice

All mice used in this study were maintained on a mixed C57BL/6-129/SvJ genetic background as described previously (14). Mice heterozygous for DS-epimerase 1 (*Dse*^+/-^) were crossed and all wild-type (WT; *Dse*^+/+^; hereafter DseWT) and knockout (*Dse*^-/-^; hereafter DseKO) littermates were used. All experiments were initiated with mice between 7–10 weeks of age. Experiments were approved by the VU University Ethical Committee according to Dutch law (MCB-14-19) or the Ethical Committee of Lund University according to Swedish law and national guidelines (M27-16).

### Fluorescent Tracers

EαGFP was a kind gift from Dr. Marc Jenkins (17) and FITC-conjugated OVA was from Thermo Fisher Scientific. Mice received an intradermal injection in the ankle. Five minutes or 40 h after injection, sdLNs were harvested, fixed as described (18), or used as single-cell suspensions after enzymatic digestion (19). ER-TR7 staining surrounding the high endothelial venules (HEVs) was surface-masked using Imaris Software (Bitplane, version 8.02 or higher). Within this mask, EαGFP mean intensity was measured.

### Quantification of DCs in Skin

Skin biopsies were pretreated (20) and processed as described (21). In short, for quantification of DCs in skin, ears of mice were fixed for 20 min in ice-cold acetone, washed with PBS, dehydrated with methanol series (20, 40, 60, 80, and 100%) twice, and subsequently rehydrated. Ears were blocked with blocking Fc-receptors using antibody clone 2.4G2 o/n at 4°C, and subsequently incubated with directly labeled primary antibody against MHCII (clone M5/114, Alexa Fluor 647 labeled) for 4 days in PBS, 4°C, rotating. After three washing steps in PBS, ears were embedded in 1.5% low-melting agarose for easy handling. Samples were dehydrated with methanol series (20, 40, 60, 80, and 100%) twice, and overnight incubated in 1:1 methanol:Benzyl-Alcohol/Benzyl-Benzoate (BABB; 1:2, both Sigma). Next morning, all solutions were replaced with BABB and stored in the dark until acquisition. Acquisition was performed using the Ultramicroscope (La Vision BioTec, Bielefeld). Images were analyzed using Imaris Software. DCs were detected using spots detection function. To calculate the minimal distance between the next spot, we used the Spots to Spots closest distance Xtension, which calculates and displays the distance to the closest neighbor of the Spots objects to determine DC density in the skin.

### DC Migration Assays

For the Transwell migration assay, DCs were isolated from spleens, using Magnisort CD11c positive selection kit (Invitrogen) according to manufacturer’s protocol. After purification, 50 × 10^3^ cells were placed in the upper compartment of a 5.0 µm pore size Transwell (Corning Costar Corp., Corning, NY, USA) and allowed to migrate for 2 h in the absence (medium only) or presence of 250 ng/mL recombinant mouse CCL21 (R&D systems) to the lower compartment. For the dermal sheet DC migration assay, ears were split into dorsal and ventral halves as described before (20, 22), and dorsal skin part was allowed to float on culture medium. Migration of cells was permitted for 40 h either or not in the presence of 250 ng/mL rmCCL21 (R&D systems). For both migration assays, total migrated cells were quantified using an LSR-Fortessa X20 (BD Biosciences) flow cytometer.

### Immunization and Tissue Isolation

Mice received an intradermal injection in the ankle of 100 µg OVA (CalBiochem) with adjuvant in 50 µL PBS (23). Blood was taken at indicated time points to determine serum immunoglobulins. Isolated spleen and LNs were snap frozen in Tissue Tek OCT (Sakura Finetek) for immunohistochemistry, or used as single cells for culture and/or flow cytometry after enzymatic digestion.

### Immunofluorescence Staining

Staining was performed as described (24). mAbs used were anti-CD11c-FITC (clone M17/4), anti-CD11c (clone N418), anti-IFNγ-APC (clone XMG1.2), B220-AF647 (clone RA3-6B2), anti-CD4-PE (clone GK1.5), anti-CD8-PE (clone 53-6.7), anti-GL7-biotin (clone GL.7), anti-CD38-PE (clone 90), goat anti-rat-AF555 all from eBioscience (ThermoFisher Scientific, MA, USA), anti-IgD-AF647 (clone 11-26c, BioLegend, CA, USA), anti-ER-TR7 (clone ER-TR7), anti-CD45-AF647 (clone MP33), and anti-MHCII-AF647 obtained from laboratory hybridomas. Biotin was detected using streptavadin-PerCP-Cy5.5 (eBioscience). Tissue was analyzed using a Zeiss fluorescent microscope (AXIO Imager.D2, Carl Zeiss) or Leica DM6000, captured with ZEN 2 pro software (version 2.0.0.0, Carl Zeiss) or Leica software and processed with Adobe Photoshop and Illustrator CS6.

### OVA-Specific ELISA

ELISA was performed as described (25). In brief, high-binding 96-well plates (Nunc Maxisorp) were coated with 5 µg/mL OVA (Sigma-Aldrich) and blocked with 2% bovine serum albumin (BSA) in PBS. Serial dilutions of serum in 1% BSA/PBS were incubated for 2 h at RT. Detection of OVA-specific antibodies was achieved by using polyclonal rabbit-anti mouse total Ig-HRP (1:2,000; Dako, P0161) in 1% BSA/PBS for 1 h at RT and subsequent incubation with 3,3′,5,5′-tetramethylbenzidine as a liquid substrate at RT for a maximum of 8 min. Absorbance was measured at 450 nm minus the absorbance at 570 nm. OVA-specific antibody titers were calculated as area under the curve, normalized to a standard, and shown as arbitrary units (a.u.).

### T Cell Restimulation

Splenocytes were isolated 10 days after immunization and restimulated in 200 µL per well (1 × 10^6^ cells/mL) with MHCII restricted OVA_262–276_ peptide (100 µg/mL) for 16 h or with MHCI restricted OVA_257–264_ peptide (0.1 µg/mL) for 5 h at 37°C, of which the final 5 h of both stimuli in the presence of GolgiPlug (BD Biosciences).

### Flow Cytometry

Cell staining was performed with mAbs as listed above after blocking Fc–receptors with antibody clone 2.4G2. OVA-specific germinal center (GC) B cells were identified using 5 µg/mL OVA-488 (Invitrogen). IFNγ was detected as described (25). Cells were acquired on a Cyan ADP (Beckman Coulter) or LSR-Fortessa X20 (BD Biosciences) flow cytometer, and analyzed with Flowjo package 10 (Tree Star). Live/dead cells were distinguished using Live-Dead-eFluor780 (eBioscience).

### Statistical Analysis

Significance was determined by GraphPad Prism performing a two-tailed unpaired Student’s *t*-test.

## Results

### *Dse*-Deficient sdLNs Display Normal Cellular Organization and Ag Collection

**Figure 1:**
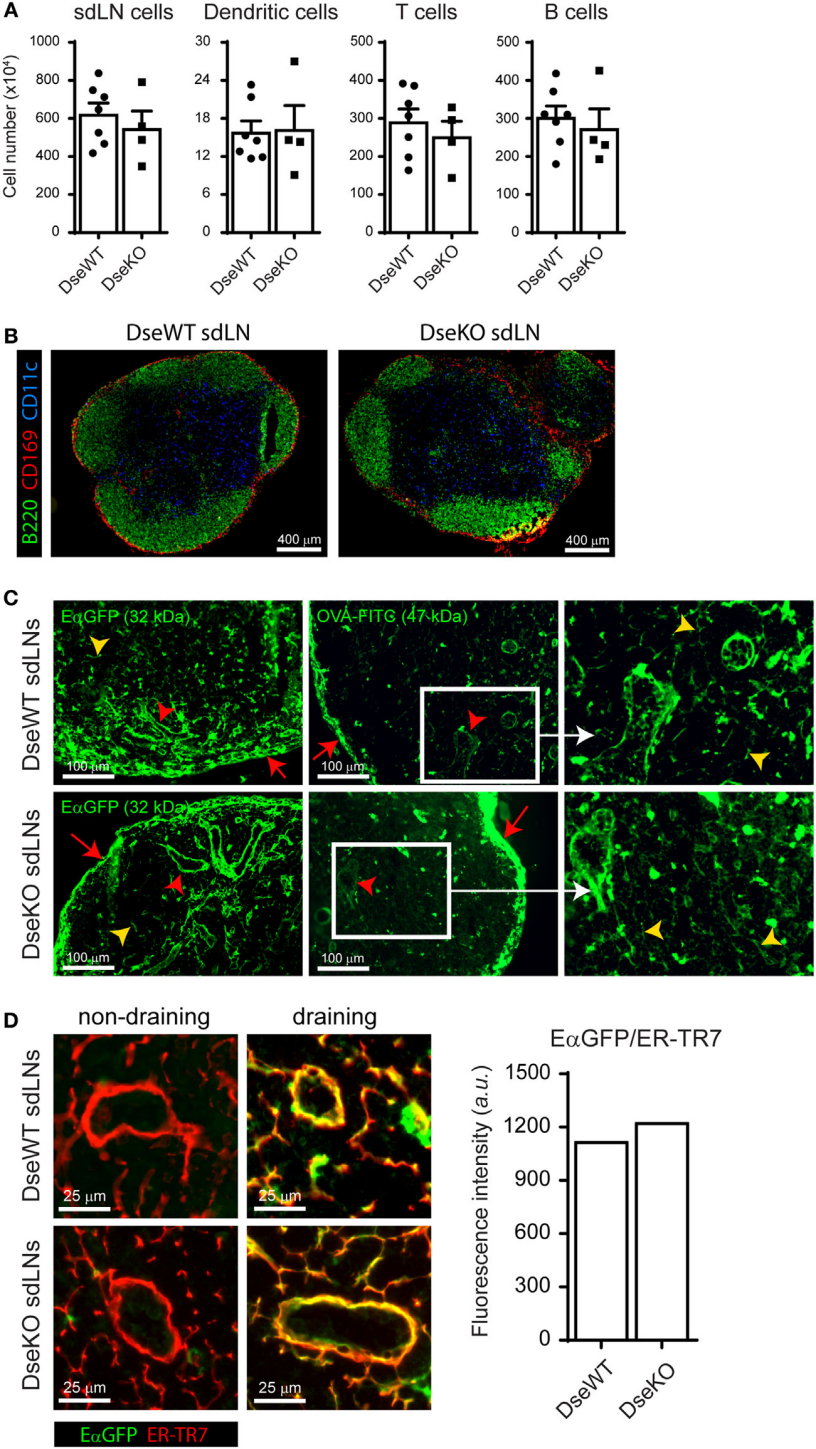
Cellular organization and antigen collection capability of skin-draining lymph nodes (sdLNs). **(A)** Total sdLN cells, CD11c+ dendritic cells (DCs), CD3+ T cells, and CD19+ B cells of wildtype (DseWT) and *Dse* deficient (DseKO) mice were quantified upon isolation of single cells from homeostatic adult sdLNs (mean ± SEM; *n* = 4–7 mice; average of four sdLNs per mouse is shown). **(B)** sdLN tissue sections of untreated adult DseWT and DseKO mice stained for B cells (B220, green), DCs (CD11c, blue), and subcapsular sinus macrophages (CD169, red). **(C)** Distribution of EαGFP (left panels) and FITC-conjugated OVA (middle panels), 5 min after intradermal injection, in the subcapsular sinus (red arrows), the conduit system (yellow arrow heads), and surrounding the high endothelial venules (red arrow heads) of sdLNs. Right panels are an enlargement of white boxes (representation of *n* = 4 mice, 2 sdLNs/mouse analyzed). **(D)** EαGFP co-localization with ER-TR7 (right images) in DseWT and DseKO sdLNs after intradermal EαGFP injection compared to the contralateral side (left images) of the same mice. Bar diagram represents the average fluorescence intensity (arbitrary units; a.u.) of EαGFP that co-localizes with ER-TR7 expression.

Impaired Ag drainage or lower cell numbers in sdLNs could affect the outcome of an adaptive immune response initiated upon intradermal immunization (1, 2). For this reason, we first set out to determine the amount of immune cells present in homeostatic LNs of adult DS-epimerase 1 (*Dse*) deficient mice and compared these with LNs of age-matched WT littermates. Quantification of single-cell suspensions by flow cytometry of sdLNs revealed equal total cell numbers, including similar amounts of total DCs, T cells, and B cells, all immune cell subsets critically involved in the initiation of an adaptive immune response (Figure 1A). In addition, fluorescent immunohistochemistry analysis showed a comparable cellular architecture as to WT LNs, with B cells organized in follicles lined by subcapsular sinus macrophages, and DCs similarly dispersed throughout the T cell zone (Figure 1B). Finally, we performed short-term (5 min) fluorescent tracer experiments to study the Ag drainage capacity of *Dse* deficient sdLNs. Notably, we observed that Ags ranging from 32 to 47 kDa readily saturated the conduits and sinuses quickly reaching the HEVs upon entering of the subcapsular sinus (Figure 1C). Indeed, as exemplified by EαGFP (Figure 1D), the intradermally injected fluorescent tracer completely co-localized with ER-TR7 expression, a marker for the conduits produced by lymphoid stromal cells (26) and surrounding HEVs. Quantification of the average EαGFP intensity within the conduits revealed no difference between WT and *Dse*-deficient mice (Figure 1D).

Together, these results demonstrate that sdLNs of *Dse*-deficient mice are equally capable of rapidly collecting and distributing soluble Ags *via* the subcapsular sinus toward the HEVs after intradermal administration and that comparable architecture and cell numbers permits equivalent Ag exposure to allow the initiation of an adaptive immune response.

### *In Vivo* DC Migration from Skin to Draining LNs Is Impaired in *Dse*-Deficient Mice

**Figure 2:**
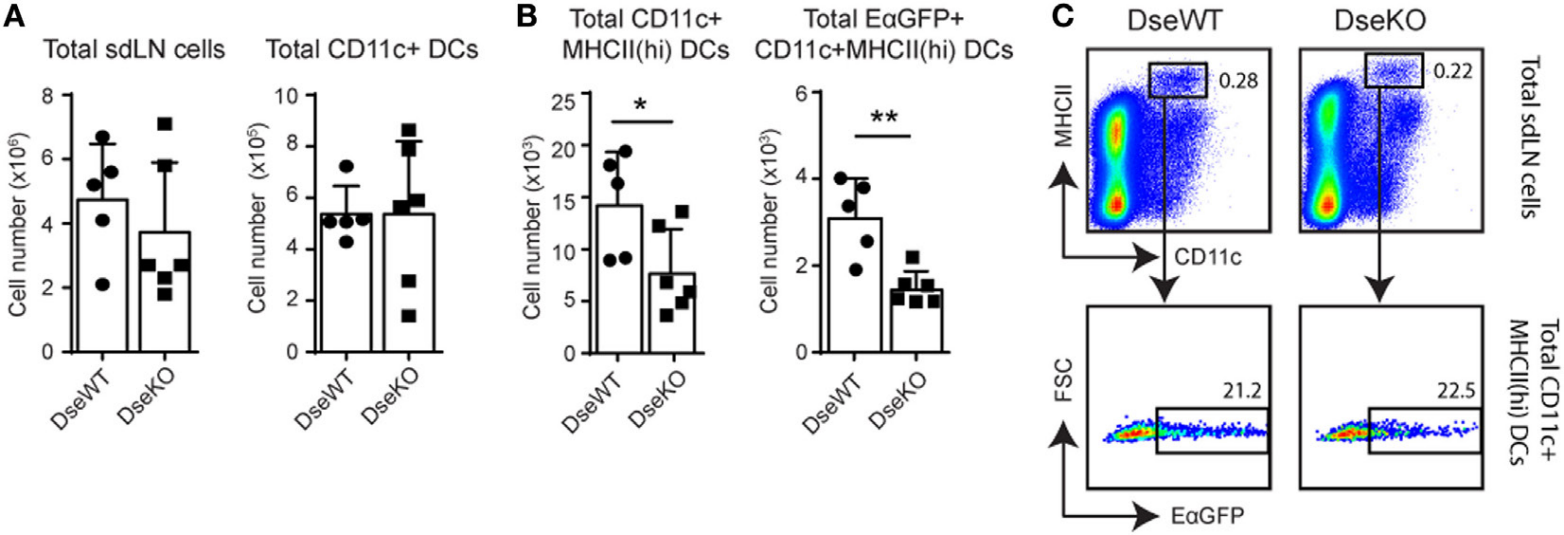
*In vivo* migration of dermal dendritic cells (DCs) to skin-draining lymph nodes (sdLNs). sdLNs of wildtype (DseWT) and *Dse*-deficient (DseKO) mice were isolated 40 h after intradermal injection with EαGFP in the ankle. **(A)** Absolute number of total sdLN cells (left panel) and CD11c+ DCs (right panel, sdLN DCs) recovered from the sdLNs 40 h after intradermal injection. **(B)** Absolute number of total MHCII(hi) DCs (left panel) and MHCII(hi)EαGFP+ DCs (right panel) found within the total CD11c+ DC population of the sdLNs after intradermal injection [for **(A,B)** bars are mean ± SD; *n* = 3 mice, 2 sdLNs/mouse, *p ≤ 0.05, **p ≤ 0.01]. **(C)** Gating strategy used to identify CD11c+ MHCII(hi)EαGFP+ migratory DCs in the sdLNs, as presented in **(A,B)**.

Antigens reach the draining LN not only as soluble particles (5, 27, 28), but a large part is also taken up by DCs in the skin for processing, transport, and presentation to LN-resident T cells (2, 28). This sequential peptide:MHC presentation of Ags to naïve T cells by DCs is essential for maximal activation of the adaptive immune response initiated in the Ag-draining LN (1, 2). Since we previously found an increase in collagen fiber diameter in the skin of *Dse*-deficient mice (14) and because skin-derived DCs need to degrade this dense collagen network in the dermis to reach the afferent lymphatics (8), we determined the *in vivo* migration efficiency of CD11c+ skin-derived DCs to the nearest draining LN (20). For this purpose, we performed intradermal injections in the ankle of mice with an Ag consisting of amino acids 46–74 of I–E^dα^ MHCII subunit fused to a green fluorescent protein (EαGFP), which allowed for easy tracing of Ag-bearing skin-derived DCs (2). Importantly, it was previously shown that after 24 h, cells that were EαGFP+ within draining LNs were almost exclusively CD11c+ MHCII(hi) expressing skin-derived DCs (2, 29). Forty hours after intradermal injection of this fluorescent tracer the total cell number, and the absolute amount of CD11c+ DCs of the skin-draining (popliteal) LN were similar (Figure 2A). However, quantification of the MHCII(hi) expressing migratory CD11c+ EαGFP+ DCs that had taken up Ag from the skin showed that more than 50% fewer Ag+ cells reached the sdLNs in *Dse*-deficient mice (Figures 2B,C). Remarkably, not only Ag+ DCs were affected in their migration to *Dse*-deficient sdLNs, also the total number of CD11c+ MHCII(hi) DCs were reduced to half of the population observed in WT littermates, irrespective of Ag uptake (Figures 2B,C). For these MHCII(hi) skin-derived DCs, it is known that they continually migrate from the skin at a low rate to the draining LN and that upon intradermal immunization the migratory numbers significantly increase (2, 30).

In summary, these findings imply that in *Dse* deficient mice both basal as well as induced migration of CD11c+ MHCII(hi) DCs from the skin to the draining LN is reduced.

### *Ex Vivo* Basal and CCL21-Induced Migration of *Dse*-Deficient DCs Is Unaffected

**Figure 3:**
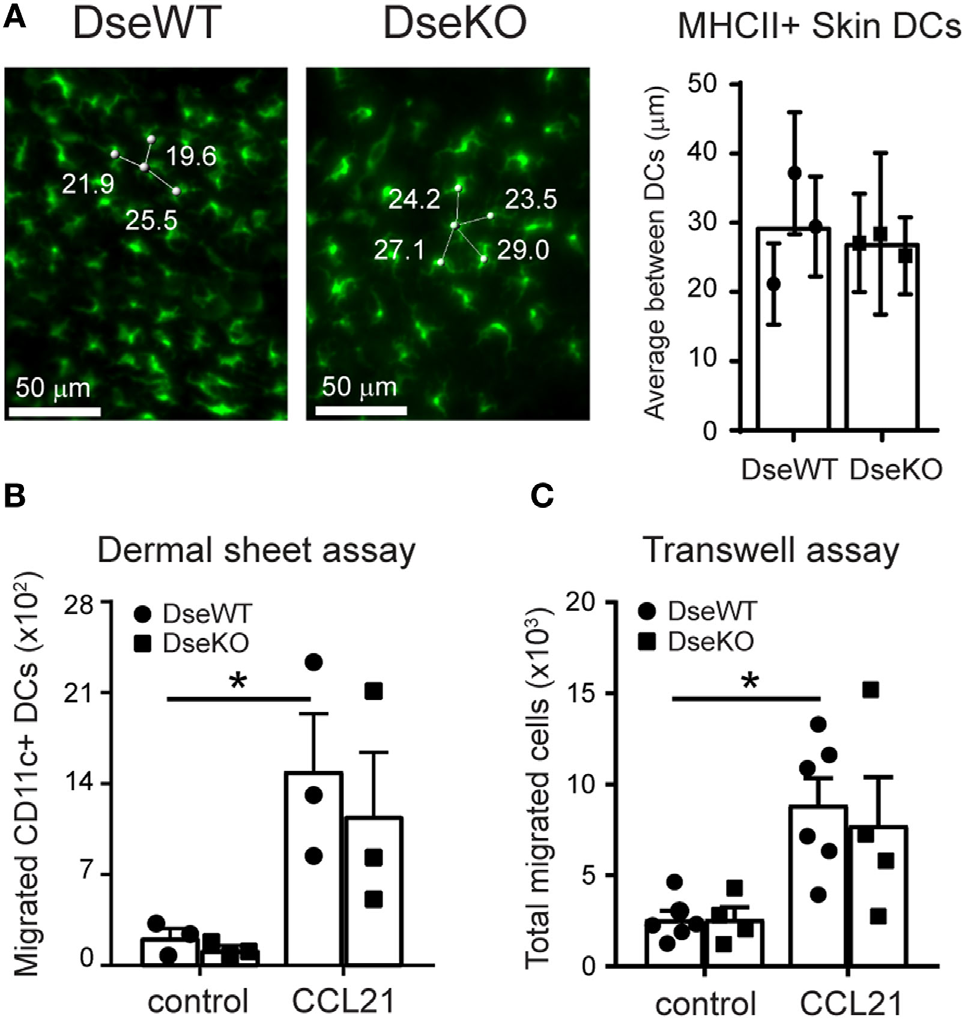
Skin distribution and *ex vivo* intrinsic migration potential of dendritic cells (DCs). **(A)** MHCII (green) expressing DC density in skin of wildtype (DseWT) and *Dse*-deficient (DseKO) mice. Three and four distances (in micrometers) are shown. The shortest distance is used (e.g., 19.6 µm) as average distance between MHCII expressing DCs and shown in the bar diagram (*n* = 3 mice, ≥40 cells/mouse analyzed throughout the whole tissue). **(B)** Total CD11c+ DCs that migrated from dermal sheets of ears (dorsal part) upon mechanical disruption obtained from DseWT and DseKO mice. Cells were allowed to migrate for 40 h either or not in the presence of CCL21 (250 ng/mL). **(C)** DCs were isolated from spleen and allowed to migrate for 2 h in the absence (control) or presence of 250 ng/mL CCL21 in a Transwell system. **(B)** Bars indicate mean ± SEM (*n* = 3 mice, one ear per condition, *p ≤ 0.05). **(C)** Bars indicate mean ± SEM of 4–6 experiments performed in triplicate (each dot represents an individual mouse, *p ≤ 0.05).

To determine whether the difference in CD11c+ MHCII(hi)EαGFP+ cells that reached the draining LNs could be explained by reduced presence of DCs in the skin of *Dse*-deficient mice, we analyzed homeostatic skin of adult WT and *Dse*-deficient littermates. By applying 3D ultra-microscopy of whole mount ears, we could clearly detect that the distribution of skin-resident MHCII expressing DCs in *Dse*-deficient mice was comparable to WT littermate controls throughout the whole tissue (Figure 3A).

Next, to address if these DCs were equally able to migrate from their location in the skin, we collected uninflamed ears and separated the dorsal from the ventral halves, exposing the dermis using forceps (20, 22). We specifically used the ears, as the dermis of the mouse ear skin is very thin, and prone to injury, which upon mechanical separation of the two halves results in the disruption of the local microenvironment, including the collagen network (31). Therefore, in contrast to other parts of the body skin, including the ankle, this skin tissue can be readily used to monitor basal and chemokine-induced *ex vivo* migration without the barrier function of the ECM. For measuring DC emigration capacity, we transferred the dorsal halves, dermis down, to a culture dish, and allowed the cells to migrate for 40 h either or not in the presence of 250 ng/mL CCL21, a potent chemoattractant and promoter of transmigration of DCs across lymphatic endothelia (32). Using this specific assay, we found that upon exposure of the dermis, the *ex vivo* migratory capacity with a disturbed ECM barrier is similar for skin-residing DCs of *Dse* deficient and WT mice, irrespective of the presence of CCL21, as comparable cell numbers were acquired (Figure 3B).

Finally, to further support equal migratory functionality, we purified DCs obtained from *Dse*-deficient and WT animals. Upon isolation, the DCs were allowed to migrate *in vitro*, toward a gradient of 250 ng/mL soluble CCL21 or medium only for 2 h, using a Transwell migration assay. Importantly, in line with the *ex vivo* dermal sheet migration assay, both basal and CCL21-induced migration of *Dse*-deficient DCs was unaffected (Figure 3C).

As such, these results highly suggest that the observed *in vivo* impaired migration of skin-derived DCs to the draining LNs in *Dse*-deficient mice is neither a result of DC seeding density in the skin, nor a reflection of intrinsic defects in the migration ability of the DCs themselves, but rather an effect of an altered ECM composition in the dermis of the intact skin.

### Loss of *Dse* Results in a Reduced Cellular Immune Response

**Figure 4:**
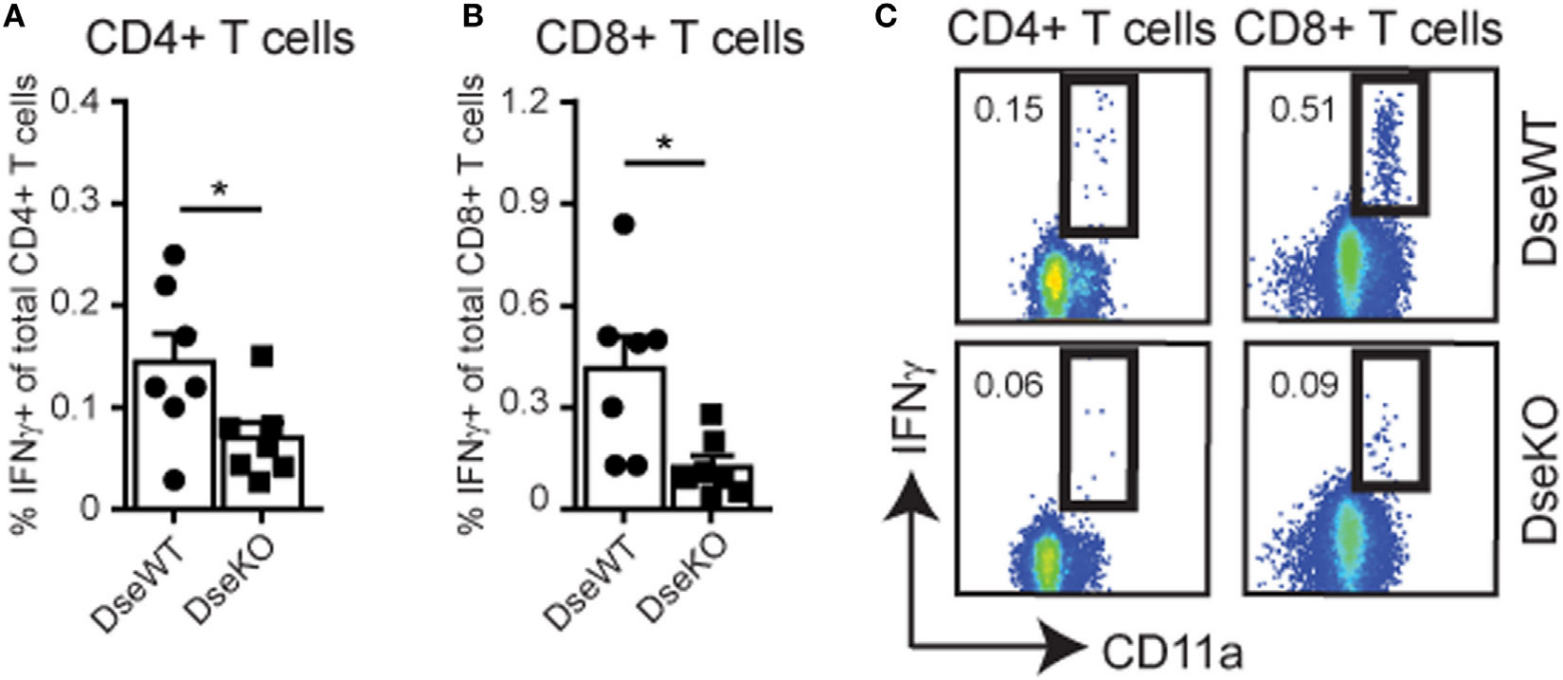
IFNγ production of OVA-specific CD4+ and CD8+ T cells. Total splenocytes of wildtype (DseWT) and *Dse*-deficient (DseKO) mice were isolated 10 days after intradermal OVA injection in the ankle, and subsequently stimulated with MHC class II restricted OVA_262–276_ peptide or with MHC class I restricted OVA_257–264_ peptide to induce the production of IFNγ in CD4+ T cells **(A)** and CD8+ T cells **(B)**, respectively, and shown as percentage of IFNγ-producing over total CD4+ or CD8+ T cells (mean ± SEM; *n* = 7 mice; *p ≤ 0.05). **(C)** Gating strategy of CD4+ and CD8+ IFNγ-producing CD3+ CD11a+T cells **(A,B)**.

Since we identified a more than 50% reduction in the amount of Ag-bearing DCs in the sdLNs upon intradermal injection (Figure 2B), we hypothesized that naïve T cell activation in sdLNs would elicit a lower response in *Dse*-deficient mice. For this purpose, we challenged mice with an intradermal injection of OVA, together with an inflammation inducing adjuvant, to study the Ag-specific cellular immune response. Ten days after immunization, mice were sacrificed and spleens (33) were collected to examine OVA-specific T cell responses by means of restimulation with OVA peptides (Figures 4A–C). Using either OVA_262–276_ peptide for CD4+ T cells or OVA_257–264_ peptide for CD8+ T cells (25), we were able to identify gamma interferon (IFNγ) production in OVA-specific CD11a+ T cells (Figure 4C). For *Dse*-deficient mice, we found that the percentage of CD4+ T cells producing IFNγ was only half of what we observed in control mice (Figures 4A,C), while the CD8+ T cell response was even diminished by more than 70% (Figures 4B,C).

This severe reduction in T cell activation implies that DS-epimerase 1 is required for controlling the efficiency of the T cell responses by regulating the amount of dermal DCs that can reach the sdLNs and present peptide:MHC complexes for further activation of initially primed T cells.

### *Dse* Deficient Mice Have Fewer Ag-Specific GC B Cells and Lower Antibody Titers

**Figure 5:**
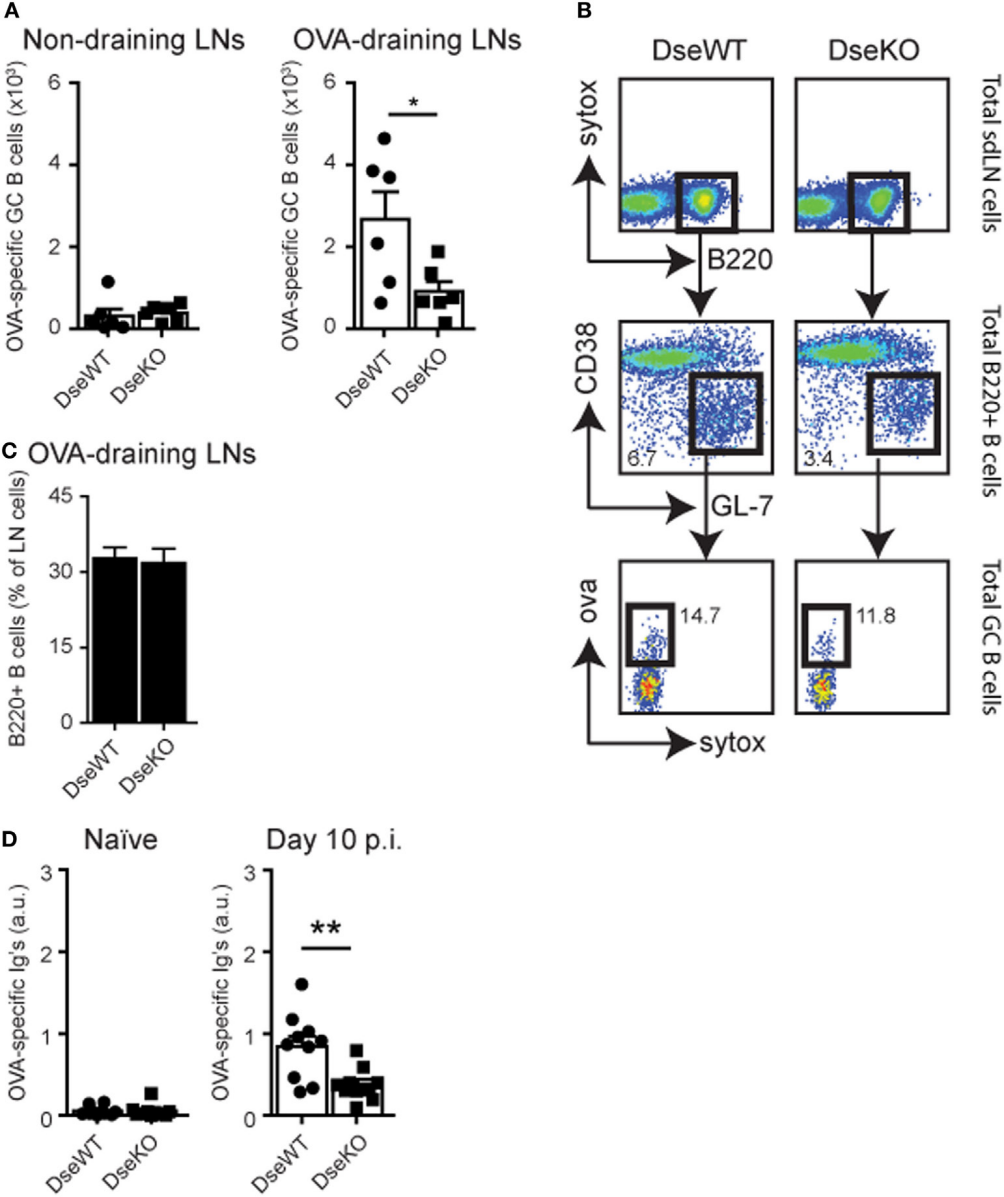
OVA-specific germinal center (GC) B cells and serum immunoglobulin levels. **(A)** Control non-draining lymph nodes (LNs) and OVA-draining LNs of wildtype (DseWT) and *Dse*-deficient (DseKO) mice were isolated 10 days after intradermal OVA challenge and analyzed for OVA-specific GC B cells (mean ± SEM; *n* = 6 mice, 2 LNs/mouse; *p ≤ 0.05). **(B)** Percentage of B220+ B cells measured in OVA-draining LNs 10 days after intradermal OVA injection. **(C)** Gating strategy used to determine OVA-specific GC B cells. **(D)** OVA-specific serum immunoglobulins (arbitrary units, a.u.) measured by ELISA before (naïve) and 10 days after immunization (mean ± SEM; *n* = 10 mice, of two independent experiments; **p ≤ 0.01).

For B cells to become Ag-specific antibody-producing plasma cells, they need to specifically recognize their Ag, and upon B cell receptor engagement, they require cognate T cell help to initiate a GC reaction (34). These cognate T cells are derived from the peptide:MHC specific CD4+ T cell pool, of which the amount of IFNγ-producing cells was severely reduced (Figures 4A,C). In line with these findings, we found that the absolute number of Ag-specific GC B cells, 10 days after intradermal OVA injection, was also more than 50% lower in *Dse*-deficient mice (Figures 5A,B). Since we found normal drainage of soluble Ag into the draining LN (Figures 1C,D), this illustrates that the limited amount of cognate helper CD4+ T cells (Figures 4A,C) impedes the induction and recruitment of normal quantities of GC B cells into the GC reaction resulting from fierce competition for peptide:MHC complexes (34, 35). As a consequence, OVA-specific immunoglobulin titers in *Dse* deficient mice measured at day 10 after immunization were much lower (<55%) compared to WT littermates, despite the detection of equal numbers of B220+ B cells in OVA-draining LNs (Figures 5C,D). This further supports the notion that fewer Ag-specific B cells have been recruited into the GC reaction, leading to less antibody-producing plasma cells and a significant reduction of OVA-specific serum immunoglobulin levels in *Dse*-deficient animals (34, 35).

Taken together, our results support an important and novel role for DS-epimerase 1 in regulating the normal release of Ag-bearing DCs from the dermis of the skin, through modification of the ECM, in particular collagen fibrillogenesis (14, 36). Accordingly, naïve T and B cells were inadequately activated in the sdLN, leading to an inefficient commencement of the subsequent Ag-specific adaptive immune response, triggered upon intradermal immunization.

## Discussion

Upon infection, a vast array of Ags intrude the host and reach the nearest collecting LNs through different mechanisms. While a proportion of Ag quickly travels *via* lymphatic vessels into the lymphatic sinuses and conduits of a draining LN (5, 27, 28), to be acquired by local DCs, a large part is taken up by DCs at the site of infection for transport to the same downstream LN (1, 2). The latter process takes more time, because DCs located in the skin need to traverse and degrade a collagen-rich layer in the dermis to reach the afferent lymphatics (8, 30). In this way, Ag encounters distinct DC subsets that in two waves present peptide:MHC complexes to T cells in the draining LN. This well-timed combination has been demonstrated to be essential for rapid, proper, and long-lasting immunity (1, 2). Notably, hybrid CS/DS chains critically participate in the collagen fibril maturation (36), and in its absence, alters the skin collagen structure (14). Here, by using mice deficient for DS-epimerase 1, an essential enzyme in CS/DS biosynthesis (13), we provide novel evidence that suboptimal synchronization of these Ag waves, caused by altered collagen fibrillogenesis resulting in thicker fibers in the dermis, has severe consequences for the initiation of an efficient adaptive immune response triggered in the skin.

As a result of *Dse* ablation, we found a consistent impairment of *in vivo* DC emigration from the skin toward the draining LN, while cell number and architecture were unaffected allowing normal distribution of equal amounts of Ag throughout the LN sinuses and conduit system, reaching the HEVs. More specifically, our findings suggest that the basal low migration of MHCII(hi)CD11c+ migratory DCs from the skin was reduced, independent of inflammation. This was further supported by our observation that once fluorescent Ag was injected, it resulted in fewer Ag+ skin-derived DCs in the draining LNs approximately 2 days after introduction into the dermis. This implies that MHCII(hi) migratory (Ag-loaded) DCs in the dermis encounter a physical barrier that is harder to breach in the absence of DS-epimerase 1 (14), leading to diminished peptide:MHC presentation in the sdLN upon Ag exposure. Although a disrupted role for Ag presentation in the sdLN by Langerhans cells (LCs) in our model cannot be entirely excluded, we argue that the effect is marginal because it was suggested that dermal DCs have an Ag uptake advantage, as intradermal injection leads to Ag deposition in the dermis (2). Nevertheless, the impaired migration of CD11c+ MHCII(hi) DCs from the skin, which includes LCs, had a major impact on the functional outcome of the Ag-specific immune response. Indeed, after intradermal immunization with OVA in combination with an inflammation inducing adjuvant, we found that both the cellular and humoral part were affected, resulting in lower numbers of Ag-specific T cells and GC B cells, and a decrease in protective serum antibody levels.

Notably, DCs have been shown to express DS-epimerase 1 to some extent, but *D4st1* transcripts were undetectable (6). This is important, since D4st1 can only generate CS/DS hybrid structures in conjunction with DS-epimerase 1, and *vice versa*. These structures are most often observed on decorin and biglycan, two important proteoglycans. Likewise, these multifunctional CS/DS proteoglycans are absent in DCs (6), while highly expressed by fibroblasts from the skin (7, 14). Overall, this suggests that DCs do not possess a suitable biosynthetic machinery to argue a DC intrinsic defect in *Dse*-deficient mice (37). Rather the results seem to reflect that altered collagen fibers in the skin are underlying the reduced DC migration that we observe here. Indeed, our data show that the *ex vivo* and *in vitro* basal and CCL21–dependent migratory capacity of DCs was unaffected by the loss of *Dse* expression, but in the absence of an ECM network with normal integrity. Altogether, this underscores our notion that the barrier function of intact skin is critically involved in the regulated release of migratory DCs through DS-epimerase 1-dependent modulation of the ECM in the dermis.

It should be pointed out that our findings are in support with results obtained in *Sparc*-deficient mice, where the amount of collagen fibers in the skin was reduced without affecting the size in diameter. This eventually led to an accelerated DC migration from the skin and a subsequent rapid priming and full activation of naïve T cells in the draining LN (9). Together with our results, these data demonstrate that spatiotemporal regulation of the composition and tightness of the ECM can have significant consequences for the adaptive immune response initiated in the skin, which calls for more in-depth analysis of the ECM in controlling the onset of immune processes. This could likely have a broader effect on immune reactions originating in other tissues consisting of balanced ECM contents, including the lung, for which we have shown high epimerase activity that is lost upon DS-epimerase 1 disruption (14).

In summary, our data show that a change in structure of CS/DS GAGs in the dermis is detrimental for the induction of an efficient adaptive immune response in sdLNs in mice. We previously demonstrated that the presence of DS-epimerase 1 is necessary for the formation of collagen fibrils of a consistent and essential size in diameter (14), which is controlled by CS/DS GAGs (10, 13), and that dysregulation of the CS/DS biosynthetic machinery crucially affects migration of Ag-loaded DCs residing in the skin. Our findings could be clinically important, as humans that lack *DSE* or *CHST14* expression have been identified (16, 38–41). These patients suffer from a connective tissue disorder known as musculocontractural Ehlers–Danlos syndrome subtype, which includes skin hyperextensibility as a result of abnormal distribution and deposition of collagen fibers in the dermis of the skin (16, 41, 42). Hence, based on our results, vaccination strategy in patients with connective tissue disorders involving the skin ECM might need reconsideration, and susceptibility to opportunistic skin infections should be explored in more detail.

## Ethics Statement

This study was carried out in accordance with the recommendations of Dutch law and Swedish law and national guidelines. The protocols were approved by the VU University Ethical Committee according to Dutch law (MCB-14-19) or the Ethical Committee of Lund University according to Swedish law and national guidelines (M27-16).

## Author Contributions

Conceptualized and supervised the study: RR. Conceived and designed the experiments: RR, RN, JK, HV, and RM. Performed the experiments: RN, RR, JK, HV, and TK. Analyzed the data: RR, RN, JK, HV, JH, AM, RM, and MM. Wrote the paper: RR, RN, MM, and RM. Provided technical assistance, essential materials, and/or feed-back: XS, AZ, JH, AM, and MM.

## Conflict of Interest Statement

The authors declare that the research was conducted in the absence of any commercial or financial relationships that could be construed as a potential conflict of interest.

## Abbreviations

CS/DS, chondroitin/dermatan sulfate; Dse/Dsel, dermatan sulfate epimerase 1/2; DC, dendritic cell; ECM, extracellular matrix; GC, germinal center; GAG, glycosaminoglycan; KO, knockout; LN, lymph node; WT, wild-type.
